# Analysis of molecular evolution of nucleocapsid protein in Newcastle disease virus

**DOI:** 10.18632/oncotarget.21373

**Published:** 2017-09-28

**Authors:** Wentao Fan, Yuliang Xu, Pu Zhang, Peng Chen, Yiran Zhu, Ziqiang Cheng, Xiaona Zhao, Yongxia Liu, Jianzhu Liu

**Affiliations:** ^1^ College of Animal Medicine and Veterinary Medicine, Shandong Agricultural University, Tai’an 271018, PR China; ^2^ Research Center for Animal Disease Control Engineering Shandong Province, Shandong Agricultural University, Tai’an 271018, PR China; ^3^ Shandong Provincial Engineering Technology Research Center of Animal Disease Control and Prevention, Shandong Agricultural University, Tai’an 271018, China; ^4^ Central Hospital of Tai’an City, Tai’an 271018, China

**Keywords:** Newcastle disease virus, bayesian phylogenetics, evolutionary rate, population dynamics, selective pressure

## Abstract

The present study investigated the molecular evolution of nucleocapsid protein (NP) in different Newcastle disease virus (NDV) genotypes. The evolutionary timescale and rate were estimated using the Bayesian Markov chain Monte Carlo (MCMC) method. The p-distance, Bayesian skyline plot (BSP), and positively selected sites were also analyzed. The MCMC tree indicated that NDV diverged about 250 years ago with a rapid evolution rate (1.059 × 10^−2^ substitutions/site/year) and that different NDV genotypes formed three lineages. The p-distance results reflected the great genetic diversity of NDV. BSP analysis suggested that the effective population size of NDV has been increasing since 2000 and that the basic reproductive number (R_0_) of NDV ranged from 1.003 to 1.006. The abundance of negatively selected sites in the NP and the mean dN/dS value of 0.07 indicated that the NP of NDV may have undergone purifying selection. However, the predicted positively selected site at position 370 was located in the known effective epitopic region of the NP. In conclusion, although NDV evolved at a high rate and showed great genetic diversity, the structure and function of the NP had been well conserved. However, R_0_>1 suggests that NDV might have been causing an epidemic since the time of radiation.

## INTRODUCTION

Newcastle disease virus (NDV) is responsible for virulent diseases in birds, thereby causing great economic losses on poultry industry [1]. NDV belongs to the *Avulavirus* genus, *Paramyxoviridae* family [2]. The paramyxoviruses have been classified into 10 subtypes, APMV-1–APMV-10 [3], and NDV belongs to APMV-1 [4]. The main structure of NDV consists of the large protein (L), the hemagglutinin-neuraminidase (HN) protein, the fusion protein (F), the matrix protein (M), the phosphoprotein (P), and the nucleocapsid protein (NP) [5]. Given their low virulence, lentogenic NDV strains cause only mild respiratory or enteric infections. By contrast, velogenic NDV strains usually cause high mortality in birds. Mesogenic isolates are intermediate-virulence NDV strains that primarily cause respiratory disease [6].

The NP primarily regulates RNA transcription, replication, and assembly. Mebatsion et al. identified that an immuno-dominant epitope on NP can be replaced or deleted by a foreign epitope. A mutation-permissive region identification on NP gene provides a potential approach to insert protective epitopes. This may be explored as target to design NDV vaccines [7]. Latest studies have reported that the NP of NDV is an important antigen in serologic assays because of its highly conserved sequences and high immunogenicity [8]. However, the molecular evolution of NP gene in NDV and whether minor antigenic changes occur in this gene remain to be elucidated. Therefore, we conducted a detailed evolutionary analysis of NP gene and predicted amino acid changes in NP three dimensional structure to provide a theoretical basis for the development of NP-based NDV vaccines.

Commonly, the genotypes and subtypes of NDV are divided into two classes, including class I (genotype I, lentogenic) and class II (contains at least genotypes II–XI, mesogenic or velogenic). Class I viruses are mainly isolated from shorebirds and waterfowl. While class II viruses are typically found spreading in poultry species and wild birds, and they have been divided into at least 10 genotypes (II–XI) [9]. Notably, NDV may evolve into virulent strain through the natural transmission between different hosts and induce major outbreaks [10]. The host shift and virulence change of NDV are closely relatedly to its evolution rate, selection pressure, and phylogenesis [11]. The pathogen’s basic reproductive number (R_0_) of a pathogen is an important epidemiological parameter that summarizes the transmission potential of a disease in a given population [12]. Despite these findings, the molecular evolution of NP gene based on genotype, evolution, and virulence has not been studied. In this study, we estimated the evolution rate, population dynamics, and selective pressure of the NP in different genotypes of NDV retrieved from public databases (characterized in our study) by the Bayesian Markov Chain Monte Carlo (MCMC) method. MCMC is a powerful algorithm for computing integrals efficiently in kinds of dimensions with one constant factor. The factor is used without parameter estimation. Therefore, MCMC offers a powerful means to compute the posterior probability density function for each model parameter in Bayesian parameter estimation [13].

## RESULTS

### Phylogenetic tree and evolutionary rate analysis of the NDV NP gene

**Figure 1:**
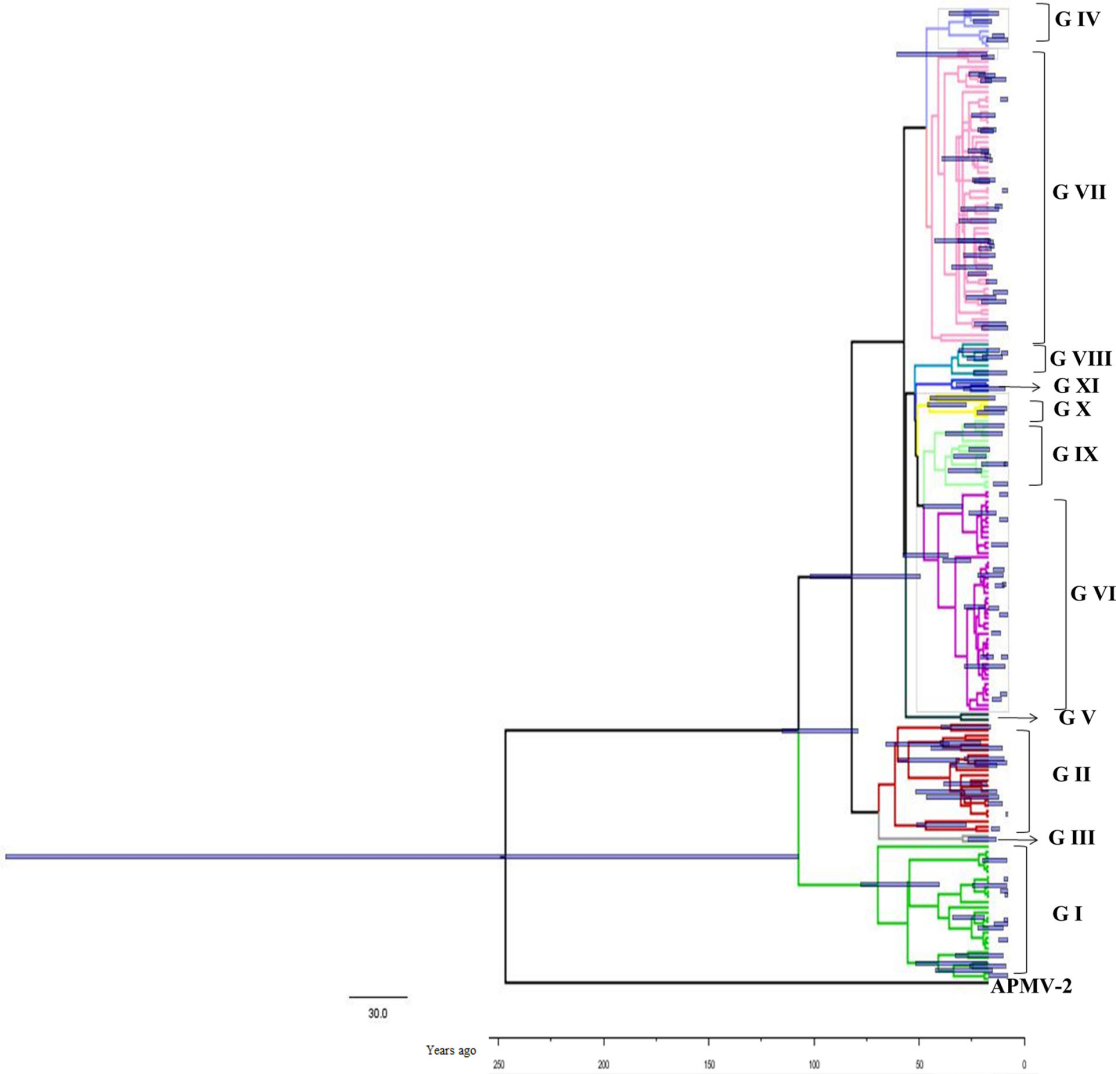
Phylogenetic tree of the nucleocapsid protein (NP) gene constructed by the Bayesian Markov chain Monte Carlo method The Markov chain Monte Carlo tree was based on the full nucleotide sequence of the NP gene (1752 nt) visualised in FigTree. Blue bars indicate 95% highest posterior density for the estimated year. The tree was estimated using an uncorrelated lognormal relaxed clock model under an exponential growth model. The scale bar represents the unit of time (year).

**Figure 2:**
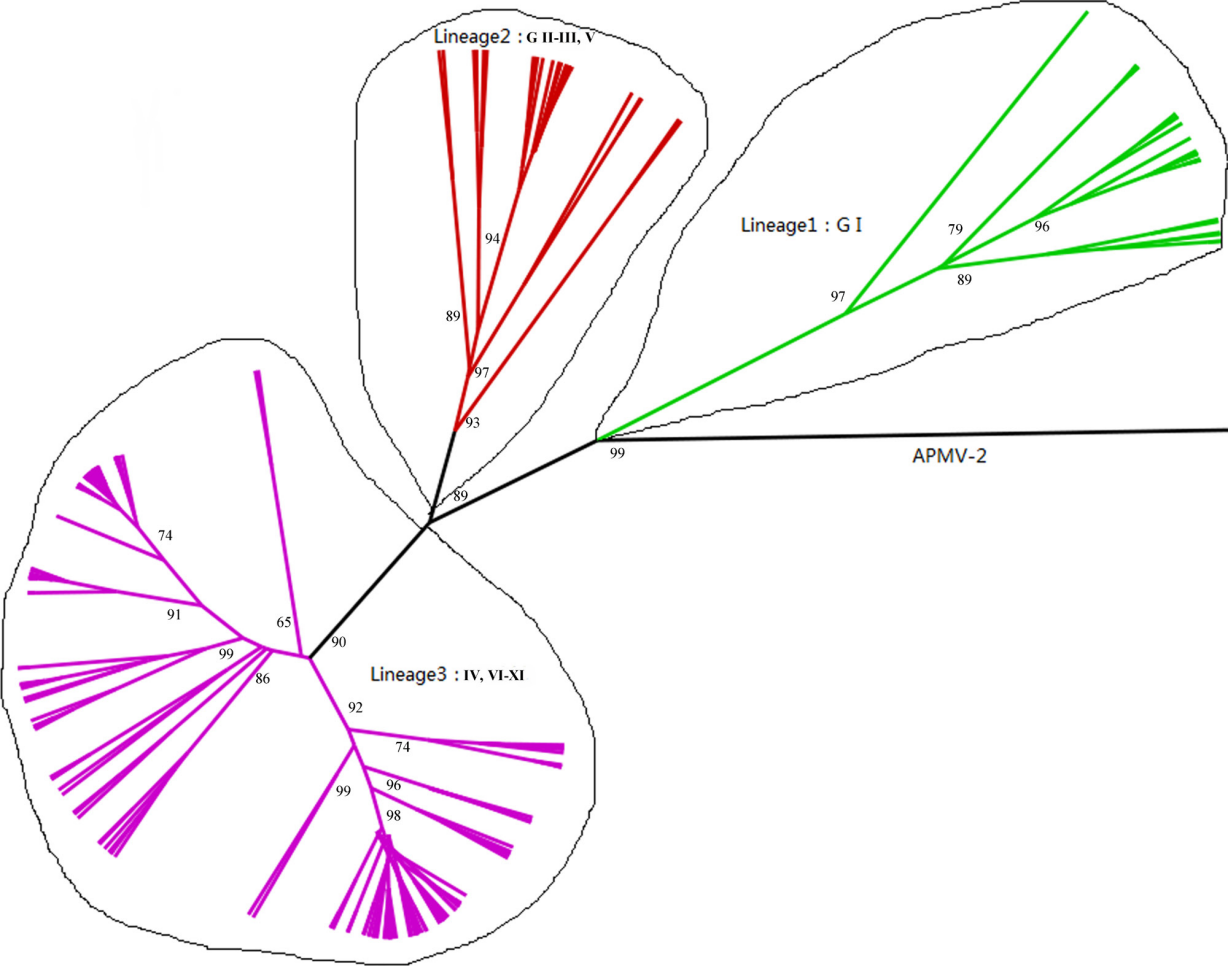
Phylogenetic tree of NP gene constructed by the ML method Labels at the branch nodes show at least 50% bootstrap support.

The time-scaled phylogenetic tree was constructed using the Bayesian MCMC method (Figure 1). The blue bars in phylogenetic tree indicate 95% highest posterior densities (HPDs) for each node. The lineages of different NDV genotype strains were estimated using a Maximum likelihood (ML) tree (Figure 2). MCMC phylogenetic tree indicated that the NDV diverged from Avian paramyxovirus 2 (APMV-2) at approximately 1766 (95% HPD 1660–1830). APMV-2 and NDV belongs to the same genus. Different genotype NDV strains formed three major lineages (lineages 1–3, Figure 2). Lineage 1 was mainly constituted by genotype I. Lineage 2 contained genotypes I, II, III, and V. Lineage 3 contained genotypes IV and VI–XI. The first major division of NDV was at the year of 1926 approximately (95% HPD 1910–1942). The divergence years of lineages 1, 2, and 3 were 1926 (95% HPD 1896–1953), 1960 (95% HPD 1945–1973), and 1970 (95% HPD 1957–1989), respectively. Different from genotype I, genotypes II–XI of NDV diverged from the same virus. The present NDV strains evolved rapidly with high evolutionary rate of 1.059 × 10 ^−2^ substitutions/site/year (95% HPD: 4.187×10^−3^ –1.74×0^−2^). Furthermore, these strains diverged approximately 100 years ago. The rapid evolution leads to at least 10 genotypes formation of class II virus.

### Genetic diversity analysis of the NDV strains

**Figure 3:**
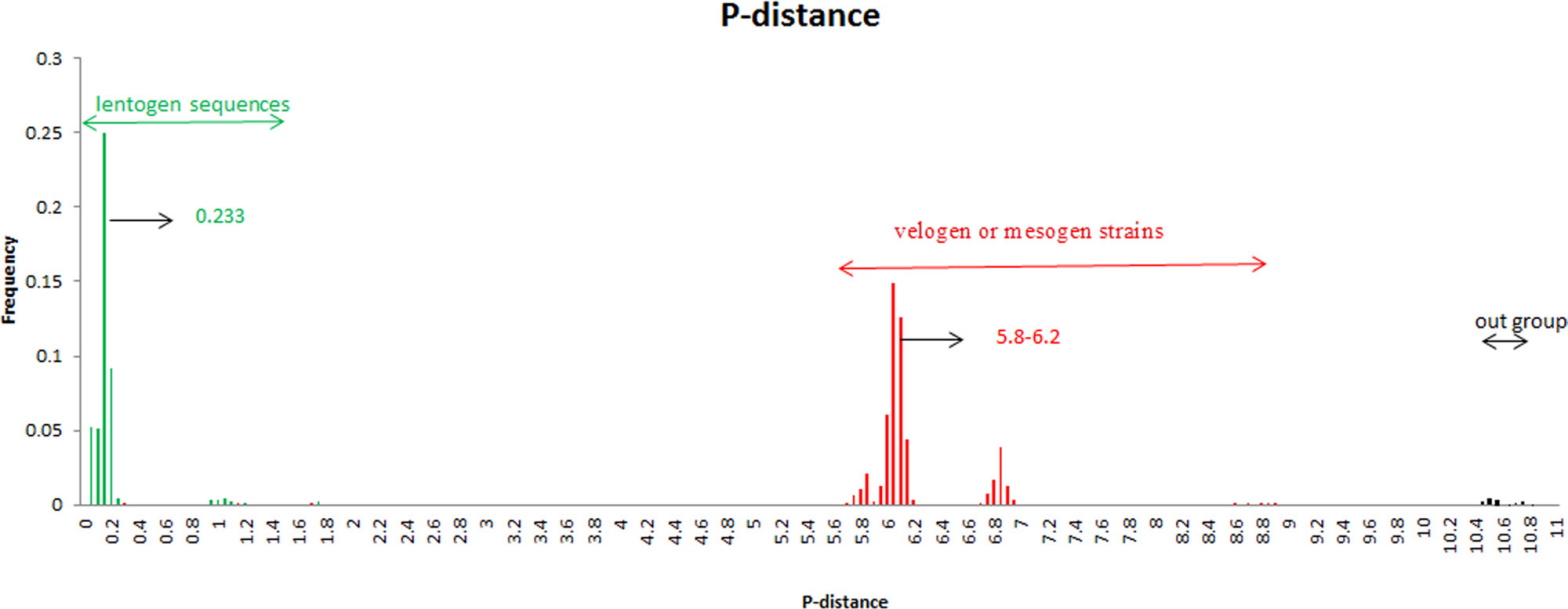
Frequency distribution of pairwise distance of NDV NP gene Green represents Class I genotype while red represents Class II genotype. A total of 170 strains were analyzed.

The collected sequences were easily divided into different genetic groups: class I (lentogenic strains) and class II (velogenic or mesogenic strains). In order to estimate the genetic distance among these NDV strains, we analyzed their pairwise distance value (p-distance). Results showed that the average p-distances within classes I and II were 0.4 and 5.09, respectively. These values were significantly lower than that between the two groups, i.e., roughly 10.79 (Figure 3). These results suggest that the NDV NP gene may be undergo great genetic divergence.

### Estimation of positive and negative selection sites of the NDV NP gene

**Figure 4:**
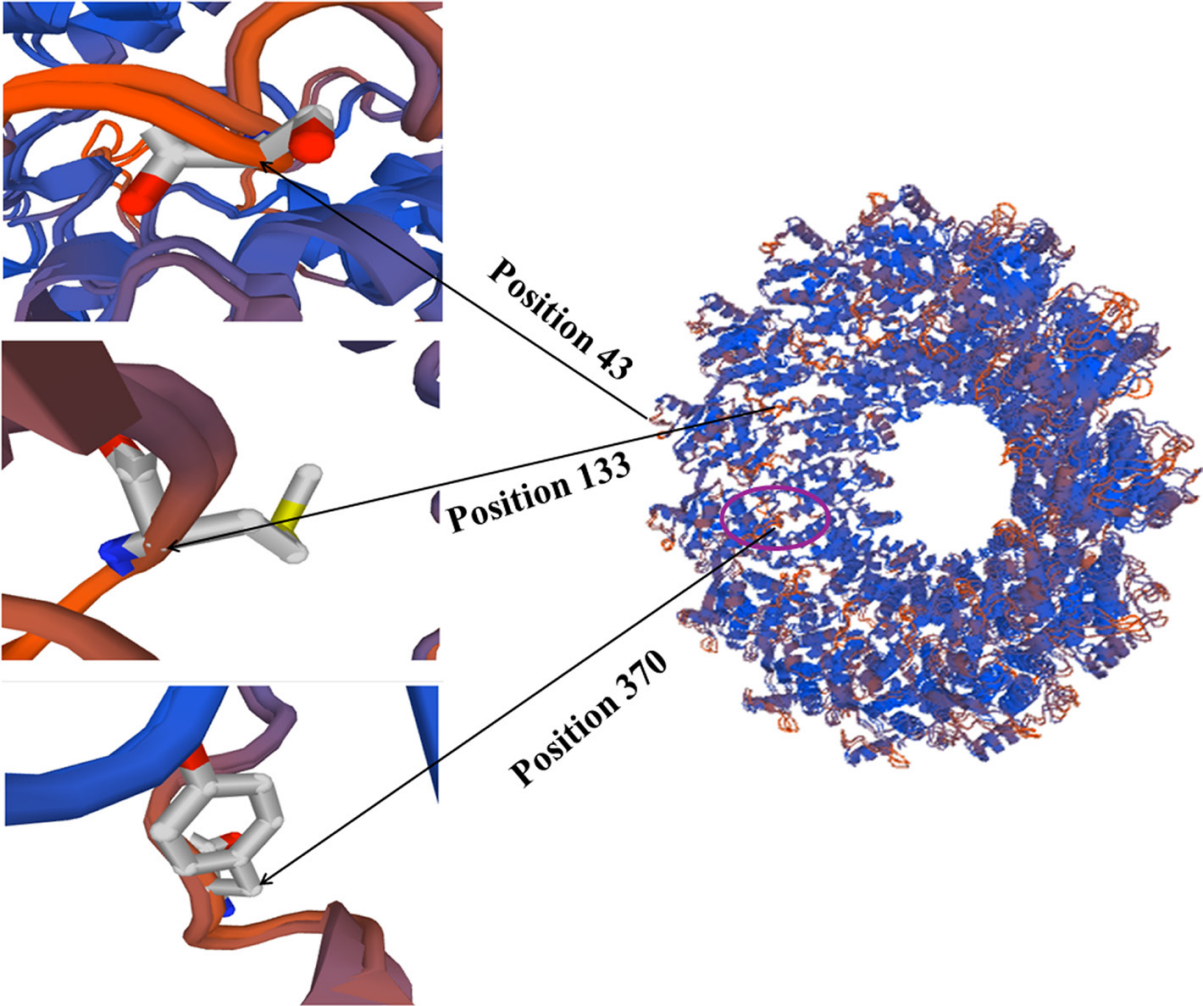
Positively selectied sites on the tertiary structure of NP protein Amino acid residues known to constitute a portion of an epitope are shown in magenta region.

The positively selected sites in NP gene of NDV were comprehensively estimated using following methods: fixed effects likelihood (FEL), conservative single likelihood ancestor counting (SLAC), mixed effects model of evolution (MEME), and internal fixed effects likelihood (IFEL). The mean dN/dS (non-synonymous rate/synonymous rate) value was estimated using the MEME method. Tables 1 and 2 show that only three positive selection sites were predicted using the FEL method and seven positive selection sites were found by the MEME method. Furthermore, all three methods indicated that most sites on the NP gene were under negative selection. A site was accepted as negative or positive selection sites if it was estimated by two methods simultaneously. Therefore, the sites at positions 43, 133, and 370 were considered positively selected. Interestingly, the site at position 370 was located in the identified epitope (aa335-aa489) region (Figure 4) [7]. The estimated mean dN/dS value of the NP gene was 0.07 (95% confidence interval 0.068–0.072). These results support that the NP protein of NDV may have undergone purifying selection due to structural and functional constraints.

**Table 1:** Positively selected sites in the NP gene

	SLAC	FEL	IFEL	MEME	dN/dS rate
	○		○	Lys4Arg	
	○		○	Ser19Pro	
	○	**Thr43Ala**	○	**Thr43Ala**	
	○	**Ala43Ser**	○	**Ala43Ser**	
	○	**Ala43Thr**	○	**Ala43Thr**	
	○	**Val133Thr**	○	**Val133Thr**	
Amino acid change	○	**Val133Met**	○	**Val133Met**	0.07
	○	**Met133Val**	○	**Met133Val**	(95%CI:0.068-0.072)
	○		○	Met151Ala	
	○	**Tyr370Ser**	○	**Tyr370Ser**	
	○		○	Lev495Pro	
	○		○	Pro495Lev	
	○		○	Gln503Pro	
	○		○	Pro503Gln	

Commonamino acid changes estimated by 4 methods are indicated in bold type. dN/dS, non-synonymous rate/synonymous rate; CI, confidence interval. Cut-off *p*-value < 0.05. ○, None positively selected sites.

**Table 2:** Negatively selected sites in the NP gene

Method	SLAC	FEL	IFEL
Number of negative selection sites	160	165	124

Cut-off *p*-value < 0.05.

### Population dynamic analysis of present NDV strains

**Figure 5:**
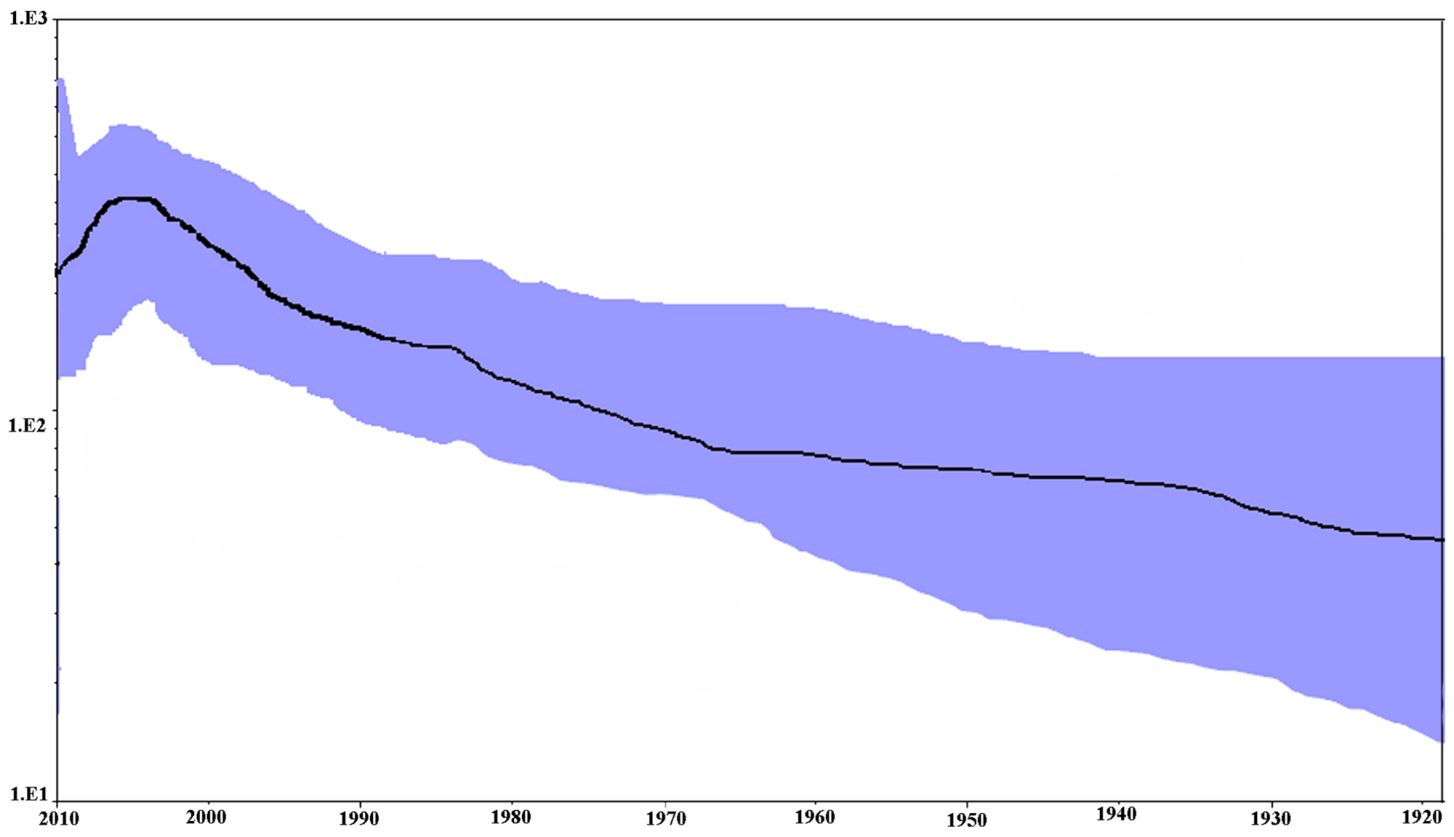
Bayesian skyline plot of NP gene in NDV The MCMC chains were run for 1,000,000 steps. The Y-axis represents the effective population size (log_10_ scale) and X-axis represents generation time (calendar year). The solid black line represents the mean value over time. The 95% HPD intervals are shown in blue region.

The population dynamics of present NDV strains based on NP gene was assessed by Bayesian skyline plot (BSP) analysis (Figure 5). Results showed that the effective population size of NDV infection number has kept relatively stable before the early 1980s but undergone a biphasic exponential growth from the 1980s to 2000s. There was one peak around the year 2000. Besides, the estimation of mean exponential growth rate was 0.144 (95% HPD 0.03–0.17) and the epidemic doubling time was about 4.8 years (credibility limits 4.0–23.1). The calculation of R_0_ for different NDV durations with rational carrier infectiousness was 1.003 for only 0.3 months’ duration to more than 1.006 for 0.5 months’ duration.

## DISCUSSION

Since the first isolation, NDV strains have been segregated into two classes that are characterized by polymorphism mainly restricted to the HN genes. However, further studies of other proteins are warranted to clarify NDV phylogenetics. The NP plays very important role in early stage of NDV life cycle. This gene is involved in the viral infection and replication so it undertakes important biologic functions. Previous studies indicated that NP could effectively induce NDV-specific antibody production in chickens [7]. Therefore, we analyzed molecular evolution of this highly conserved gene of NDV in the known genotypes (I–XI).

Results of time-scaled evolutionary tree showed that NDV diverged approximately 250 years ago. The first division of present NDV genotypes into three lineages was dating back to 1926 (95% HPD 1910–1942). The divergent years of lineages 1, 2, and 3 were 1926 (95% HPD 1896–1953), 1960 (95% HPD 1945–1973), and 1970 (95% HPD 1957–1989), respectively (Figures 1 and 2). According to previous reports, there were three pandemics of NDV and led to enormous economic losses in the world. The first panzootic started in Southeast Asia in the mid-1920s and it took about 30 years to spread to other regions [14]. The second panzootic of NDV might also originate in Asia, however it took less years than the first panzootic to conquer the same territory in the 1960s [15]. It was mainly introduced from Indonesia to the United States and Europe. Straight after, the third NDV panzootic broke out in England in the 1970s and affected chickens and racing pigeons [16]. Furthermore, Toyoda et al. [17] screened over 200 NDV strains and compared physical maps of these strains. They found that these NDV isolates, circulating before the 1970s, could be divided into three major genetic groups. The present NDV strains were estimated to evolve rapidly with mean evolutionary rate of 1.059 × 10 ^−2^ substitutions/site/year by Bayesian MCMC method (95% HPD:4.187×10^−3^ –1.74×10^−2^). The value is as high as that of highly variable RNA viruses. In a previous estimation of the evolutionary rates of NDV, Miller [18] reported that the rates for the F genomes of low virulence are 2.28 × 10^− 4^ (strict) and 2.92 × 10^− 4^ (relaxed) while the rates for virulent F genomes are 1.32 × 10^− 3^ (strict) and 1.70 × 10^− 3^ (relaxed). The high evolutionary rates may accelerate for viruses containing the virulent phenotypes. In addition, the average p-distances within class I (lentogenic strains) and class II (velogenic or mesogenic strains) were 0.4 and 5.09, respectively. The intergenogroup p-distance value was 10.79, which indicates that NDV had a wide genetic divergence (Figure 3). In fact, class I and class II are mainly divisions of NDV strains in the world and these promote extensive genetic diversities among poultry. Latest reports showed that nine distributed worldwide genotypes were further divided in waterfowls in class I strains. By contrast, class II strains has comprised 18 genotypes when they are isolated over time [9, 19]. Deserve to be mentioned, the typically circulating viruses in poultry species and wild birds belong to class II strains and genotypes IV–VIII are the predominant genotypes in worldwide poultry [20].

We used four methods in the NDV evolutionary selection pressure analysis. Although SLAC method is usually used for large alignments analysis compared with the other three methods, it detects selected sites at external branches of phylogenetic tree. In comparison, IFEL is more intensive for investigating selected sites along internal branches. Furthermore, the two methods take both nonsynonymous and synonymous rate variations into account in estimation process and could be efficiently parallelized [21]. Meanwhile, MEME can estimate episodic selective pressure [22]. The ratio of substitution rates at non-synonymous and synonymous sites is a common criterion to evaluate the evolutionary selection pressures on a protein. In general, if the normalized rate of nonsynonymous substitutions (dN) is greater than the normalized rate of synonymous substitutions (dS), that is dN/dS>1, the analyzed protein is considered under positive selection pressure. Conversely, if dN/dS<1, the protein is considered under negative selection and evolves slowly [23, 24]. Negative selection often removes deleterious mutations in biological functional protein to maintain the long-term stability during evolution [25]. The mean dN/dS ratio of 0.07 and a large amount of negative selection sites demonstrated that the NP gene has undergone purifying selection, during which disadvantageous nonsynonymous mutations in NDV have been eliminated from the population (Tables 1 and 2). Miller also reported that value corresponding to the NP gene was 0.074. As a rule, the negative selection pressure on protein may prevent the functional deterioration of viral NP. The NP gene displayed strongly negative selection probably due to NP are involved in NDV replication and this biological function needs to be relatively stable. This phenomenon was also observed in conserved regions of NDV M protein [6].

Although NPs of paramyxoviruses are relatively conserved, the monoclonal antibody reactivity could be detected antigenic differences among isolated NDV strains [8, 26]. NP of negative-strand RNA virus possesses strong immunogenic in nature. It has been used for diagnosing NDV, measles, rabies, and vesicular stomatitis virus as the antigen for several years [7]. Generally, a strong antigenicity protein of virus may undergo high levels of selection pressure, which can lead to an abundant of positive selection sites appear in antigenic viral protein [27]. In the present research, three positive selection sites were predicted in the deduced NP protein of NDV. Importantly, the amino acid at position 370 was located in the known effective epitope region of the NP (Figure 4). Thus the amino acid substitution at position 370 probably influence the host immunity efficacy against NDV. This phenomenon is in line with other viral systems that the special region of a protein may show higher rate of nonsynonymous substitutions than that of synonymous substitutions regardless of the general dN/dS ratio. Although few positively selected sites were identified for the NDV NP gene and insufficient evidence indicated the amino acid substitutions on effective epitope region substantially improve fitness of NDV *in vivo*, these analysis results may explain the reason of immunity failure of NDV vaccines.

Recombination detection showed no evidence of recombination among present NDV NP strains. Consequently, these nucleotide sequences were used for generating phylogenetic trees to conduct population dynamic analysis. Our demographic analysis showed that NDV grew at a rate of 0.114 year^−1^, and the R_0_ ranged from 1.003 to more than 1.006 due to different durations of plausible poultry carrier infectiousness of NDV. This result indicates that NDV might have caused epidemics since the time of their radiation. Furthermore, BSP result suggested that the effective population size of NDV infection number kept relatively stable until the early 1980s but underwent biphasic exponential growth from the 1980s to 2000s (Figure 5). This observation suggested that the number of genetically distinct but related NDV strains was expanding during these time. In fact, a relationship is found between epidemics of NDV and the effective population size. Many studies have reported NDV outbreaks in Brazil, Kazakhstan, and Pakistan since 1998 [28-30]. Moreover, the NDV that caused epizootics in many countries including Germany, the Netherlands, Belgium, Italy, and Spain in the late 1980s has been classified into a novel genotype, namely genotype VII. Thus, genotype VII NDV possibly originated from the Far East [31].

In conclusion, the present NDV including at least 11 genotypes formed three different linages due to rapid evolution and wide genetic divergence. The NP is considered relatively conserved among isolates of different virus genotypes. Although most positions were under purifying selection, a few positions located in specific regions were subject to positive selection. Furthermore, NDV has undergone an exponential population expansion, which could cause outbreaks in the host. These findings help us comprehensively understand the molecular evolution of NDV and prevent the spread of NDV by enhanced active surveillance.

## MATERIALS AND METHODS

### Strains and alignments

In this study, sequences of more than 98 % homologies were removed from present dataset. A total of 170 strains were collected.169 full-length sequences coding for the NP of different genotype NDV strains were collected. Furthermore, APMV-2 strain (England 7702, GenBank accession No. HM159993) was added to the dataset as the outgroup. All collected sequences are available in GenBank. Clustal W is used to align all sequences [32]. The nucleotide sequences correspond to positions 1–1752 of the NP gene in NDV strain (Pakistan/2010/Uppsala, GenBank accession No. JN682209). Supplementary Table 1 showed the detailed information of present NDV strains.

### Phylogenetic analysis by the bayesian MCMC method

The Bayesian MCMC method in BEAST software v1.7.5 was used to analyze the time-scaled phylogenetic tree and evolutionary rate of NDV [33, 34]. The suitable evolutionary model that corresponds with our data was chosen by JModelTest software v2.1.7 [35]. As a result, HKY model was previously selected as the best nucleotide substitution model [36]. In our study, we tested four clock models (random, exponential, lognormal, and strict models) and two demographic models (exponential growth and constant size). Akaike’s information criterion (AICM) were calculated to compare the clock and demographic models through MCMC [37] using Tracer software v1.6 (http://beast.community/tracer). After the comparison, we chose the model with the lowest AICM value (Supplementary Table 2). Consequently, the sequences were analyzed using an HKY model under an uncorrelated lognormal relaxed clock and an exponential growth model. R_0_ was calculated through the equation R_0_ = rD + 1, where r is the exponential growth rate and D is the average durations of rational carrier infectiousness [38]. The D used for present NDV R_0_ calculation was 0.3–0.5 month. Then, the relation λ= ln(2)/r was used to calculate the epidemic doubling time [39]. The MCMC chains were run for 10,000,000 steps and sampled every 10,000 steps. The effective sample size (ESS) after a 10% burn-in was considered to assess the convergence by Tracer software v1.6. Only parameters with ESS values of >200 were accepted. LogCombiner (available in BEAST package) combined different BEAST runs from each file after a 10% burn-in. The estimation uncertainty was indicated by showing 95% highest posterior density (HPD) intervals. Tree Annotator software v 1.7.4 generated tree with the maximum clade credibility after a 10% burn-in and FigTree software v1.3.1 was used to view the phylogenetic tree. Moreover, a BSP analysis was proceeded to estimate changes of effective population size of NDV through the time using BEAST software.

### P-distance values calculation

In order to analyze the phylogenetic relationships and genotypes definition of NDV, the frequency/p*-*distance values of the intergenotypes and outgroup were calculated. In total of 170 strains were analyzed using MEGA 6.0 software [40].

### Positive and negative selection sites estimation

To comprehensively analyze the selection pressure on the NP of NDV, we calculated nonsynonymous (dN) and synonymous (dS) substitution rates at every codon by four methods: FEL, SLAC, MEME, and IFEL. We employed more than one method for accurate calculations through Datamonkey [41]. Positive selection sites (dN>dS) and negative selection sites (dN<dS) were ascertained by the *p*-value of < 0.05. The tertiary structure model of the NDV NP was built by SWISS MODEL [42], and the positively selected sites were showed on a tertiary structure. Notable, the Genetic Algorithm Recombination Detection method [43] was employed to detect recombination of used NDV strains through Datamonkey. In consequence, the dataset included non-recombinant sequences.

## SUPPLEMENTARY MATERIALS TABLES





## Abbreviations

(NP): nucleocapsid protein
(NDV): Newcastle disease virus
(MCMC): Markov chain Monte Carlo
(BSP): Bayesian skyline plot
(R0): Reproductive number
(ND): Newcastle disease
(P): Phosphoprotein
(M): Matrix protein
(F): Fusion protein
(HN): Hemagglutinin-neuraminidase
(L): Largeprotein
(SLAC): Single likelihood ancestor counting
(FEL): Fixed effects likelihood
(IFEL): Internal fixed effects likelihood
(MEME): Mixed effects model of evolution
(CI): Confidence interval
(BSP): Bayesian skyline plot
(RNPs): Ribose nuclear proteins
(HPD): Highest posterior densities

## References

[1] Zhu W, Dong J, Xie Z, Liu Q, Khan MI (2010). Phylogenetic and pathogenic analysis of Newcastle disease virus isolated from house sparrow (Passer domesticus) living around poultry farm in southern China. Virus Genes.

[2] Yue H, Deng S, Yang FL, Li DF, Fu AJ, Yang F, Tang C (2009). Short hairpin RNA targeting NP mRNA inhibiting Newcastle disease virus production and other viral structural mRNA transcription. Virus Genes.

[3] Miller PJ, Afonso CL, Spackman E, Scott MA, Pedersen JC, Senne DA, Brown JD, Fuller CM, Uhart MM, Karesh WB, Brown IH, Alexander DJ, Swayne DE (2010). Evidence for a new avian paramyxovirus serotype 10 detected in rockhopper penguins from the Falkland Islands. J Virol.

[4] Alexander DJ (2000). Newcastle disease and other avian paramyxoviruses. Rev Sci Tech.

[5] Han GZ, He CQ, Ding NZ, Ma LY (2008). Identification of a natural multi-recombinant of Newcastle disease virus. Virology.

[6] Seal BS, King DJ, Meinersmann RJ (2000). Molecular evolution of the Newcastle disease virus matrix protein gene and phylogenetic relationships among the paramyxoviridae. Virus Res.

[7] Mebatsion T, Koolen MJ, de Vaan LT, de Haas N, Braber M, Römer-Oberdörfer A, van den Elzen P, van der Marel P (2002). Newcastle disease virus (NDV) marker vaccine: an immunodominant epitope on the nucleoprotein gene of NDV can be deleted or replaced by a foreign epitope. J Virol.

[8] Silva KR, Goncalves MC, Oliveira ES, Fernando FS, Montassier MD, Fernandes CC, Tamanine MD, Borzi MM, Santos RM, Mendonca AD, Reischak D, Paulillo AC, Montassier HJ (2014). Cloning and Expression of the Nucleoprotein Gene (NP) of Newcastle Disease Virus (NDV) in Escherichia coli for Immunodiagnosis Application. Int J Poult Sci.

[9] Kang Y, Xiang B, Yuan R, Zhao X, Feng M, Gao P, Li Y, Li Y, Ning Z, Ren T (2016). Phylogenetic and Pathotypic Characterization of Newcastle Disease Viruses Circulating in South China and Transmission in Different Birds. Front Microbiol.

[10] Dortmans JC, Koch G, Rottier PJ, Peeters BP (2011). A comparative infection study of pigeon and avian paramyxovirus type 1 viruses in pigeons: evaluation of clinical signs, virus shedding and seroconversion. Avian Pathol.

[11] Mollentze N, Biek R, Streicker DG (2014). The role of viral evolution in rabies host shifts and emergence. Curr Opin Virol.

[12] Longdon B, Hadfield JD, Day JP, Smith SC, McGonigle JE, Cogni R, Cao C, Jiggins FM (2015). The causes and consequences of changes in virulence following pathogen host shifts. PLoS Pathog.

[13] Larget B, Simon DL (1999). Markov chain Monte Carlo algorithms for the Bayesian analysis ofphylogenetic trees. Mol Biol Evol.

[14] Ballagi-Pordány A, Wehmann E, Herczeg J, Belák S, Lomniczi B (1996). Identification and grouping of Newcastle disease virus strains by restriction site analysis of a region from the F gene. Arch Virol.

[15] Lancaster JE (1976). A History of Newcastle Disease with Comments on its Economic Effects. Worlds Poult Sci J.

[16] Alexander DJ, Wilson GW, Russell PH, Lister SA, Parsons G (1985). Newcastle disease outbreaks in fowl in Great Britain during 1984. Vet Rec.

[17] Toyoda T, Sakaguchi T, Hirota H, Gotoh B, Kuma K, Miyata T, Nagai Y (1989). Newcastle disease virus evolution. II. Lack of gene recombination in generating virulent and avirulent strains. Virology.

[18] Miller PJ, Kim LM, Ip HS, Afonso CL (2009). Evolutionary dynamics of Newcastle disease virus. Virology.

[19] Snoeck CJ, Owoade AA, Couacy-Hymann E, Alkali BR, Okwen MP, Adeyanju AT, Komoyo GF, Nakouné E, Le Faou A, Muller CP (2013). High genetic diversity of Newcastle disease virus in poultry in West and Central Africa: cocirculation of genotype XIV and newly defined genotypes XVII and XVIII. J Clin Microbiol.

[20] Miller PJ, Decanini EL, Afonso CL (2010). Newcastle disease: evolution of genotypes and the related diagnostic challenges. Infect Genet Evol.

[21] Kosakovsky Pond SL, Frost SD (2005). Not so different after all: a comparison of methods for detecting amino acid sites under selection. Mol Biol Evol.

[22] Murrell B, Wertheim JO, Moola S, Weighill T, Scheffler K, Kosakovsky Pond SL (2012). Detecting individual sites subject to episodic diversifying selection. PLoS Genet.

[23] Padhi A, Ma L (2015). Time-dependent selection pressure on two arthropod-borne RNA viruses in the same serogroup. Infect Genet Evol.

[24] Dasmeh P, Serohijos AW, Kepp KP, Shakhnovich EI (2014). The influence of selection for protein stability on dN/dS estimations. Genome Biol Evol.

[25] Kushibuchi I, Kobayashi M, Kusaka T, Tsukagoshi H, Ryo A, Yoshida A, Ishii H, Saraya T, Kurai D, Yamamoto N, Kanou K, Saitoh M, Noda M (2013). Molecular evolution of attachment glycoprotein (G) gene in human respiratory syncytial virus detected in Japan 2008-2011. Infect Genet Evol.

[26] Rabu A

[27] Nielsen R (2005). Molecular signatures of natural selection. Annu Rev Genet.

[28] Fernandes CC, Varani AM, Lemos EG, de Miranda VF, Silva KR, Fernando FS, Montassier MF, Montassier HJ (2014). Molecular and phylogenetic characterization based on the complete genome of a virulent pathotype of Newcastle disease virus isolated in the 1970s in Brazil. Infect Genet Evol.

[29] Bogoyavlenskiy A, Berezin V, Prilipov A, Usachev E, Lyapina O, Levandovskaya S, Korotetskiy I, Tolmacheva V, Makhmudova N, Khudyakova S, Tustikbaeva G, Zaitseva I, Omirtaeva E (2005). Molecular characterization of virulent Newcastle disease virus isolates from chickens during the 1998 NDV outbreak in Kazakhstan. Virus Genes.

[30] Lindh E, Ekkommonen C, Alasaari J, Vaheri A, Vapalahti O, Huovilainen A (2012). Molecular Epidemiology of Outbreak-Associated and Wild-Waterfowl-Derived Newcastle Disease Virus Strains in Finland, Including a Novel Class I Genotype. J Clin Microbiol.

[31] Lomniczi B, Wehmann E, Herczeg J, Ballagi-Pordány A, Kaleta EF, Werner O, Meulemans G, Jorgensen PH, Manté AP, Gielkens AL, Capua I, Damoser J (1998). Newcastle disease outbreaks in recent years in western Europe were caused by an old (VI) and a novel genotype (VII). Arch Virol.

[32] Larkin MA, Blackshields G, Brown NP, Chenna R, McGettigan PA, McWilliam H, Valentin F, Wallace IM, Wilm A, Lopez R, Thompson JD, Gibson TJ, Higgins DG, MA L (2007). Clustal W and Clustal X version 2.0. Bioinformatics.

[33] Drummond AJ, Rambaut A (2007). BEAST: bayesian evolutionary analysis by sampling trees. BMC Evol Biol.

[34] Bouckaert R, Heled J, Kühnert D, Vaughan T, Wu CH, Xie D, Suchard MA, Rambaut A, Drummond AJ (2014). BEAST 2: a software platform for Bayesian evolutionary analysis. PLoS Computational Biology.

[35] Posada D (2009). Selection of models of DNA evolution with jModelTest. Methods Mol Biol.

[36] Tanabe AS (2011). Kakusan4 and Aminosan: two programs for comparing nonpartitioned, proportional and separate models for combined molecular phylogenetic analyses of multilocus sequence data. Mol Ecol Resour.

[37] Suchard MA, Weiss RE, Sinsheimer JS (2001). Bayesian selection of continuous-time Markov chain evolutionary models. Mol Biol Evol.

[38] Pybus OG, Drummond AJ, Nakano T, Robertson BH, Rambaut A (2003). The epidemiology and iatrogenic transmission of hepatitis C virus in Egypt: a Bayesian coalescent approach. Mol Biol Evol.

[39] Pybus OG, Charleston MA, Gupta S, Rambaut A, Holmes EC, Harvey PH (2001). The epidemic behavior of the hepatitis C virus. Science.

[40] Tamura K, Stecher G, Peterson D, Filipski A, Kumar S (2013). MEGA6: Molecular Evolutionary Genetics Analysis version 6.0. Mol Biol Evol.

[41] Delport W, Poon AF, Frost SD, Kosakovsky Pond SL (2010). Datamonkey 2010: a suite of phylogenetic analysis tools for evolutionary biology. Bioinformatics.

[42] Arnold K, Bordoli L, Kopp J, Schwede T (2006). The SWISS-MODEL workspace: a web-based environment for protein structure homology modelling. Bioinformatics.

[43] Kosakovsky Pond SL, Posada D, Gravenor MB, Woelk CH, Frost SD (2006). GARD: a genetic algorithm for recombination detection. Bioinformatics.

