# Supplementary material for: Analysis of molecular evolution of nucleocapsid protein in Newcastle disease virus
Source: Oncotarget. 2017 Sep 28;8(57):97127–36. doi: 10.18632/oncotarget.21373 (PMC5722550; doi:10.18632/oncotarget.21373)
Supplement: Supplementary file 1 [file oncotarget-08-97127-s001.pdf]

Analysis of molecular evolution of nucleocapsid protein in Newcastle disease virus

SUPPLEMENTARY MATERIALS

Supplementary Table 1: Newcastle Disease Virus NP gene cDNA sequences used in present study. (out)= sequence formally used as outgroup representatives for the NDV ingroup.

See Supplementary File 1

Supplementary Table 2: Models compared by AICM

**Model**		**AICM (SE)**
Clock model	Strict clock	1762.6 (±1.352)
	**Uncorrelated lognormal relaxed clock**	**1571.8 (±1.616)**
	Uncorrelated exponential relaxed clock	1839.7 (±0.925)
	Random local clock	1863.4 (±0.796)
Demographic model	Constant size	1398.5 (±1.642)
	**Exponential growth**	**1376.7 (±0.986)**

Data and models with bold letters are used in this study.
AICM, Akaike’s information criterion through MCMC; SE, Standerd error.
